# SenSkin™: a human skin-specific cellular senescence gene set

**DOI:** 10.1007/s11357-025-01568-y

**Published:** 2025-02-25

**Authors:** Saranya P. Wyles, Grace T. Yu, Clarisse Ganier, Tamar Tchkonia, Magnus D. Lynch, George A. Kuchel, James L. Kirkland

**Affiliations:** 1 Department of Dermatology, Mayo Clinic, 200 First Street SW, Rochester, MN USA; 2 Mayo Clinic Medical Scientist Training Program, Mayo Clinic, Rochester, MN USA; 3 Immunology Department, Metaorganism Unit, Institut Pasteur, Paris, France; 4 Center for Gerotherapeutics, Division of Endocrinology and Metabolism, Department of Medicine, Cedars-Sinai Medical Center, Los Angeles, CA USA; 5 Centre for Gene Therapy and Regenerative Medicine, King’s College London, Guy’s Hospital, London, UK; 6 St. John’s Institute of Dermatology, King’s College London, Guy’s Hospital, London, UK; 7 UConn Center on Aging, University of Connecticut, Farmington, CT USA

**Keywords:** Cellular senescence, Bioinformatics, Skin

## Abstract

Cellular senescence gene sets have been leveraged to overcome the inadequate sensitivity or specificity of single markers. However, growing evidence of heterogeneity among tissues in senescent cell phenotypes and gene expression profiles has highlighted the need for tissue-specific gene sets. SenSkin™ was curated by an expert review of literature on cellular senescence in the skin and characterized with pathway analysis. To validate SenSkin™, it was evaluated for enrichment with chronological aging in a bulk RNA-sequencing (RNA-seq) dataset and a pseudobulk RNA-seq dataset. Further, changes to SenSkin™ in different skin cell types with photoaging were evaluated in two single-cell RNA-seq datasets. SenSkin™ predominantly included genes related to the senescence-associated secretory phenotype (SASP), which were associated with metabolism and multiple aspects of immune responses. SenSkin™ was more enriched in chronologically aged skin than other commonly used cellular senescence and aging gene sets. In scRNA-seq, SenSkin™ displayed significant upregulation due to photoaging in ten skin cell types. In conclusion, SenSkin™ is a human skin-specific senescence gene set validated in chronological aging and photoaging, which may be more effective at detecting senescent cells in the skin than non-tissue-specific gene sets.

**Supplementary Information:**

The online version contains supplementary material available at 10.1007/s11357-025-01568-y.

## Introduction

As a pillar of aging, cellular senescence has been implicated in skin aging and numerous skin pathologies [1]. Cellular senescence is a defense mechanism triggered by intrinsic stressors, such as telomere attrition, or extrinsic stressors, such as UV damage. It is defined by a set of characteristics, including essentially irreversible cell cycle arrest, resistance to apoptosis, a senescence-associated secretory phenotype (SASP), and dysfunction of multiple organelles, such as lysosomes and mitochondria.

Because senescent cells have a complex phenotype with multiple affected pathways, single markers have not provided sufficient sensitivity or specificity to accurately identify senescent cells. As such, high-dimensional data, such as transcriptomics and other -omics, have been leveraged. By postulating that the accumulation of smaller changes in large numbers of genes better defines cellular senescence than any single marker, multiple cellular senescence gene sets have been developed through literature review and curation of tens to hundreds of genes that have been demonstrated to be associated with cellular senescence. Recent examples include CellAge, SenMayo, and Reactome’s Cellular Senescence gene set [2–5].

Although senescent cell phenotypes and markers have been demonstrated to vary by organ and tissue type, few if any tissue-specific cellular senescence gene sets have been developed. Therefore, we developed SenSkin™, a human skin-specific cellular senescence gene set. We applied SenSkin™ to bulk RNA-seq and scRNA-seq data generated from human skin samples. We specifically selected representative datasets of chronologically aged and photoaged skin, since these conditions are associated with higher burdens of senescent cells, to validate whether SenSkin™ can effectively detect cellular senescence in the skin. To our knowledge, SenSkin™ is the first reported skin-specific gene set for cellular senescence.

## Methods

### RNA-seq datasets

Kuehne et al.’s publicly available bulk RNA-seq dataset (GSE85358 on Gene Expression Omnibus) was used to evaluate chronological aging [6]. Epidermal skin samples from suction blisters produced on the inner forearm were sequenced, with 24 young female donors (20–25 years old) and 24 older female donors (55–66 years old).

Two publicly available scRNA-seq datasets were used to analyze changes with photoaging. Solé-Boldo et al.’s scRNA-seq dataset (GSE130973) of UV-protected inguino-iliac skin [7] was integrated and compared to Ganier et al.’s scRNA-seq dataset (E-MTAB-13085 on ArrayExpress) of UV-exposed facial skin from 15 donors (56 to 90 years old) [8]. All pre-processing, data integration, and analyses were performed in R v4.3.2 and Seurat v5.0.1 (www.satijalab.org/seurat). As previously described, datasets were combined, pre-processed, integrated, scaled, and log-normalized [8]. Unsupervised clustering of cells was performed with the first 20 PCA dimensions and a resolution of 0.65. Dimension reduction was performed using UMAP with default parameters and 20 PCA dimensions for visualization. Extra-articular chondrocytes from a full-thickness ear sample were removed as they are not typically considered skin cells. Differential gene expression for different clusters or subsets of cells was evaluated using Wilcoxon rank-sum tests with a fold change cutoff of 0.25 (natural log scale) and a Bonferroni-adjusted *p*-value cutoff of 0.05. Cell types were described based on independently established markers of human skin cell types.

### Pathway enrichment analysis

To identify pathways overrepresented by SenSkin™ genes, a Web-based Gene set analysis toolkit (WebGestalt 2024 release; www.webgestalt.org) was used. Inputs were represented by lists of gene symbols, and they were analyzed for overrepresentation against the *Homo sapiens* genome reference set. Pathways were obtained from the non-redundant biological process functional database from Gene Ontology. An FDR threshold of 0.05 was used to define significance.

### Functional enrichment analysis

STRING (v11.5, www.string-db.org) was used to identify protein–protein interaction networks and clustering patterns among the SenSkin™ genes. The highest confidence level (0.900) was employed, and standard settings were used. DBSCAN clustering was performed with standard settings (epsilon = 3).

### Hierarchical clustering analysis

Hierarchical clustering of SenSkin™ genes was performed based on SenSkin™ gene expression in the integrated scRNA-seq datasets, used as a pseudobulk RNA-seq dataset. The pseudobulk RNA-seq dataset was used instead of the bulk RNA-seq dataset because all SenSkin™ genes were sequenced in it, compared to 145 out of the 165 genes in the bulk RNA-seq dataset. Distributions of expression of individual genes were determined, and because nine of the SenSkin™ genes had a skewness greater than 2, gene expression was log-transformed. Next, the expression of each gene was standardized according to a Gaussian distribution, and correlations between genes were calculated. The corrplot v0.95 package was used to perform complete linkage clustering, and after manual tuning, five clusters were evaluated and visualized in a heatmap.

### Gene set enrichment analysis

Gene set enrichment analysis (GSEA v4.3.3, www.gsea-msigdb.org) was performed on MSigDB (v2024.1) with default settings, specifically, 1000 permutations on phenotypes with no collapse. Younger and older samples from Kuehne et al. were compared in the evaluation of the H hallmark collection, CP: REACTOME collection, CellAge, GenAge, and SenMayo [2–5, 9]. An FDR threshold of 0.25 was used to define significance.

### Single-cell gene scoring

To evaluate the overall expression of the SenSkin™ gene set for each cell, a composite score based on a normalized sum of *z*-scores was used. After gene expression values were log-normalized, they were standardized to each gene, yielding *z*-scores. SenSkin™ genes that were expected to be upregulated in cellular senescence (164 genes) were separated from those that were expected to be downregulated (1 gene: *LMNB1*). The sum of *z*-scores for the genes expected to be upregulated in cellular senescence was calculated and normalized by the number of genes (164) and then subtracted by the normalized sum of *z*-scores for the genes expected to be downregulated (1) to produce the composite score.

### Statistical analysis

Violin plot statistics were performed using Wilcoxon signed-rank tests with stat_compare_means() from ggpubr v0.6.0. Other specific statistical analyses are described in the corresponding method sections.

## Results

### Development and characterization of a skin-specific cellular senescence gene set, SenSkin™

SenSkin™ was developed by a literature review of genes associated with cellular senescence in human skin [5, 7, 10–14]. Two board-certified dermatologist-scientists with research laboratories in skin biology conducted the review of peer-reviewed articles, and the genes were categorized into those that were upregulated in senescent skin cells (164 genes) and those that were downregulated in senescent skin cells (1 gene, *LMNB1*), comprising a total of 165 genes (shown in Table 1). SenSkin™ includes 18 genes in common with SenMayo, 36 genes in common with CellAge, 19 genes in common with GenAge, and 13 genes in common with Reactome’s Cellular Senescence pathway (shown in Fig. S2) [2–5].

**Table 1:** SenSkin™ genes by gene symbols

A2M	CTSL	KLF6	RASD1	TRIB1
ADAMTS1	CTSZ	KRT15	RGS16	TSKU
ADAMTS4	DDR1	KRT18	RNASET2	UBC
ADAMTS9	DEC1	LAMA5	RND3	VCAM1
ADAMTSL4	DUSP1	LGALS3	RNF122	VEGFA
ANGPTL4	DUSP5	LMNB1	RPS3	VEGFC
ANPEP	EGFL7	LOX	SAT1	YBX3
ANXA2	EGR1	LTBP1	SBNO2	ZC3H12A
APOE	EIF4A1	MAFF	SEMA3F	ZFP36
APOLD1	EMP1	MAN2B1	SEMA4B	
AQP1	ETS2	MAPK11	SERPINB1	
ARID5A	F3	MCL1	SERPINE1	
B2M	FOS	MIDN	SERPING1	
BCL3	FOSL1	MOV10	SERTAD1	
BCL6	FOSL2	MT1A	SFN	
BHLHE40	GADD45A	MT1M	SHC1	
BTG2	GADD45B	MT1X	SLC25A25	
C10orf10	GLIPR1	MYC	SLC2A3	
C11orf96	GPR4	NAMPT	SLC39A1	
C1QTNF1	HAPLN3	NEDD9	SLC39A14	
C1RL	HILPDA	NFIL3	SLCO4A1	
C1S	ICAM1	NFKB2	SNAI1	
C3	IER2	NFKBIZ	SNRPC	
CASP4	IER3	NR4A1	SOCS3	
CCNL1	IFI16	NUCB1	SOD2	
CD14	IGFBP2	NXT1	STAT3	
CD59	IGFBP3	OSMR	STC1	
CD63	IGFBP4	PDGFB	SUSD6	
CDK2	IGFBP7	PDLIM1	TGFBI	
CEBPB	IL1R1	PIM1	TGIF1	
CEBPD	IL4R	PLAUR	THBD	
CFB	IL6	PLK3	THBS1	
CHSY1	INHBB	PLSCR1	TIMP1	
CLEC3B	ITPKC	PLTP	TIMP3	
CREM	ITPRIP	PNP	TNFRSF10B	
CRISPLD2	JUN	PNRC1	TNFRSF10D	
CSF1	JUNB	PPP1R18	TNFRSF12A	
CSRNP1	KIAA0040	PPRC1	TNFRSF1A	
CTSB	KLF10	PROS1	TP53	

**Fig. 1:**
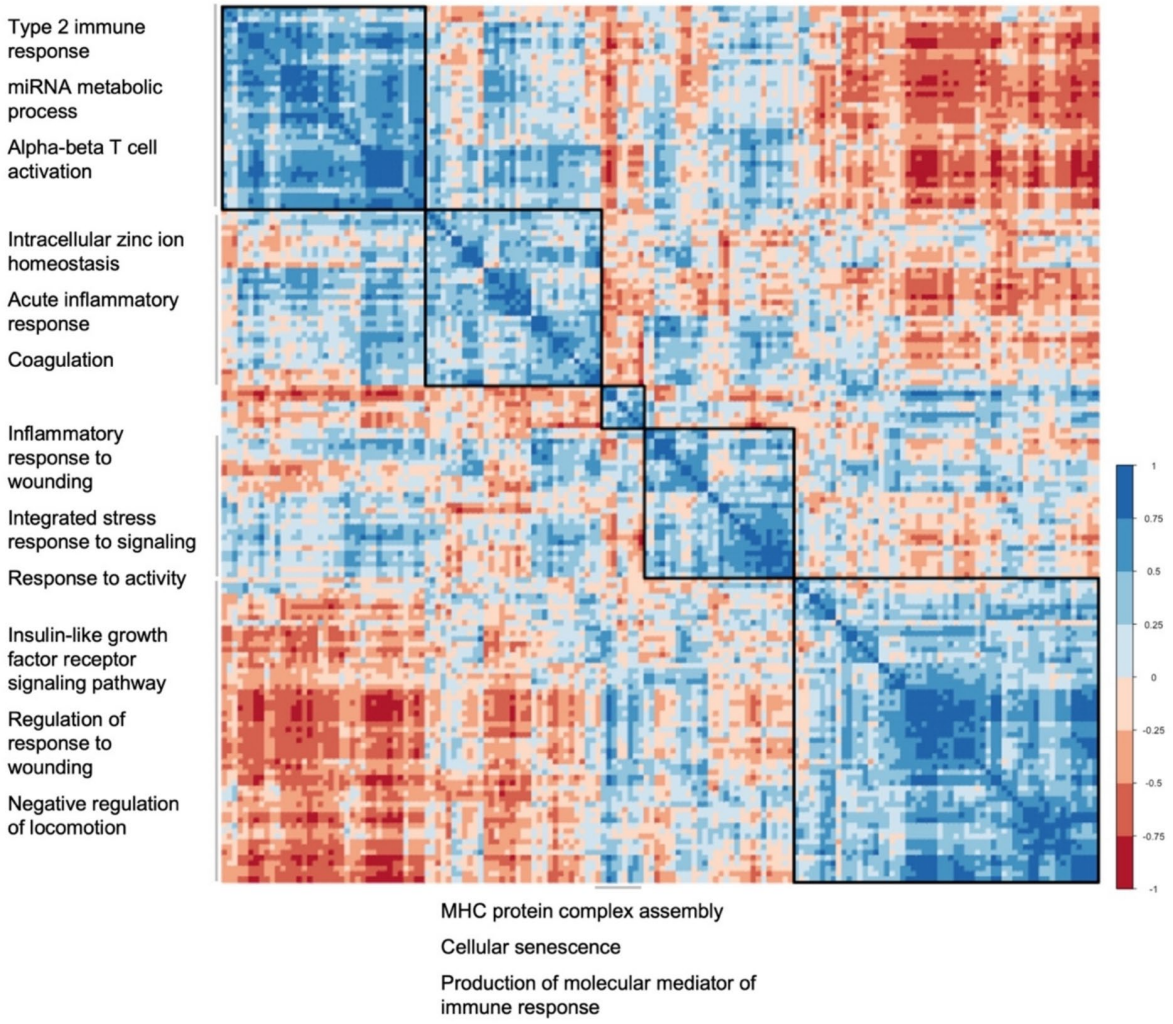
Hierarchical clustering of SenSkin™ genes based on inter-gene correlations. Complete linkage hierarchical clustering of SenSkin™ genes based on correlations between pairs of genes in integrated pseudobulk RNA-seq datasets. The top three pathways represented by each cluster are listed

To identify pathways captured by SenSkin™, genes were analyzed altogether, and then, groups of highly correlated genes were analyzed as distinct groups. Many of the top Gene Ontology biological process pathways overrepresented by SenSkin™ genes as a whole were related to wound repair and immune responses, such as connective tissue development, mononuclear cell differentiation, wound healing, and regulation of hemopoiesis (shown in Fig. S1a). These pathways are consistent with findings that senescent cells affect cutaneous wound healing and modulate the immune system [15, 16]. Next, genes were hierarchically clustered based on correlations between the expression of pairs of genes in the integrated scRNA-seq datasets, and the pathways represented by each cluster were delineated (shown in Fig. 1). By examining clusters of highly correlated genes, overall mechanisms could be observed with less chances of focusing on individual genes with counter-regulatory mechanisms or genes that are associated but not directly involved in cellular senescence. Clusters of genes in SenSkin™ represent stress response, innate immunity, adaptive immunity, insulin-like growth factor pathway, and antigen presentation.

Genes in SenSkin™ were clustered based on known protein–protein interactions to evaluate the landscape of pathways captured by SenSkin™ (shown in Fig. S1b). The largest cluster of genes was identified as being related to cellular senescence, demonstrating concordance between SenSkin™ and other cellular senescence gene sets. Other clusters were related to features of cellular senescence, including altered lipid metabolism, immune responses, and TGF-β pathways.

### High enrichment of SenSkin™ in bulk RNA-seq of chronological skin aging

**Fig. 2:**
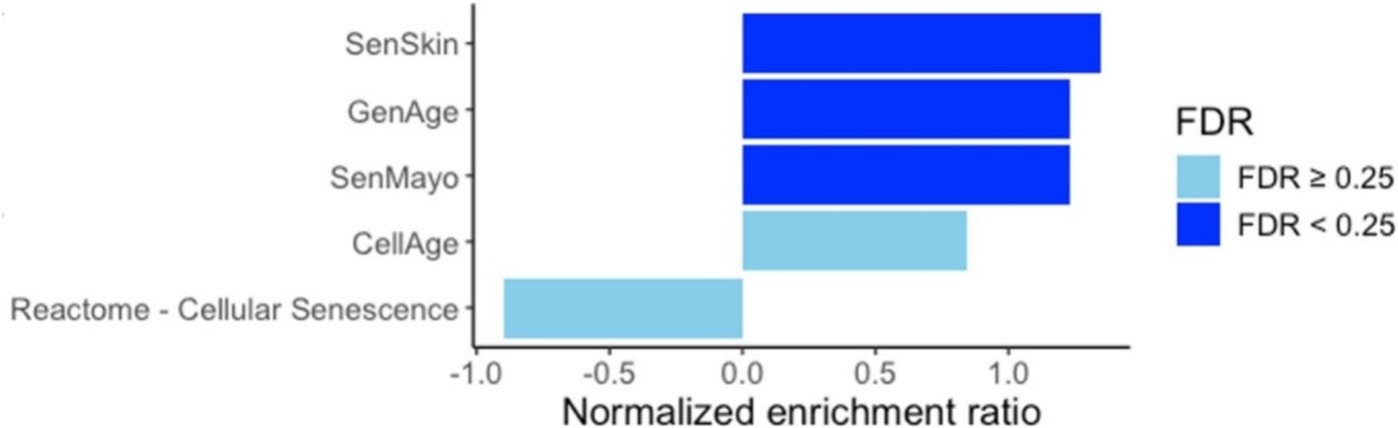
Enrichment of SenSkin™ in chronological aging in RNA-seq of epidermis. Gene set enrichment analysis normalized enrichment ratio of SenSkin™ and four commonly used cellular senescence gene sets. FDR < 0.25 is considered significant

To evaluate the ability of SenSkin™ to detect senescent cell accumulation with chronological aging, SenSkin™ was used to analyze bulk RNA-seq. Samples from 24 younger (20 to 25 years old) donors were compared to 24 older (55 to 66 years old) donors from Kuehne et al.’s dataset of normal human epidermis from the inner forearm [6]. Gene set enrichment analysis revealed that SenSkin™ was significantly enriched in samples from older patients, with a normalized enrichment score of 1.34 (shown in Fig. 2 and Fig. S3a).

Gene set enrichment analysis of SenSkin™ was compared to three cellular senescence gene sets (CellAge, SenMayo, and Reactome’s Cellular Senescence pathway) and one age-related gene set (GenAge) (shown in Fig. S3a-e). Of the five gene sets evaluated, three were significantly enriched in the older subjects’ samples: SenSkin™, GenAge, and SenMayo. This finding suggests that SenSkin™ effectively detected changes related to chronological aging in the skin while some of the other general cellular senescence gene sets could not, namely CellAge and Reactome’s Cellular Senescence pathway. Moreover, SenSkin™ had the highest normalized enrichment score, suggesting that SenSkin™ was more associated with chronological skin aging than other cellular senescence or aging gene sets. Although Kuehne et al.’s dataset only included epidermal tissue, SenSkin™ also had the highest normalized enrichment score when evaluating chronological aging in Solé-Boldo et al.’s dataset of full-thickness human skin as pseudobulk RNA-seq (shown in Figure S3f).

### Elevated SenSkin™ score with photoaging in majority of cell types in skin scRNA-seq

To evaluate the use of SenSkin™ in scRNA-seq and photoaging, scRNA-seq datasets from Solé-Boldo et al. and Ganier et al. were integrated [7, 8]. Because the samples were obtained from UV-protected inguino-iliac skin and UV-exposed facial skin respectively, the effects of UV exposure were evaluated as an established method of cellular senescence induction. A composite single-cell gene set score based on the normalized sum of *z*-scores was used to calculate enrichment in SenSkin™ as previously described [17].

**Fig. 3:**
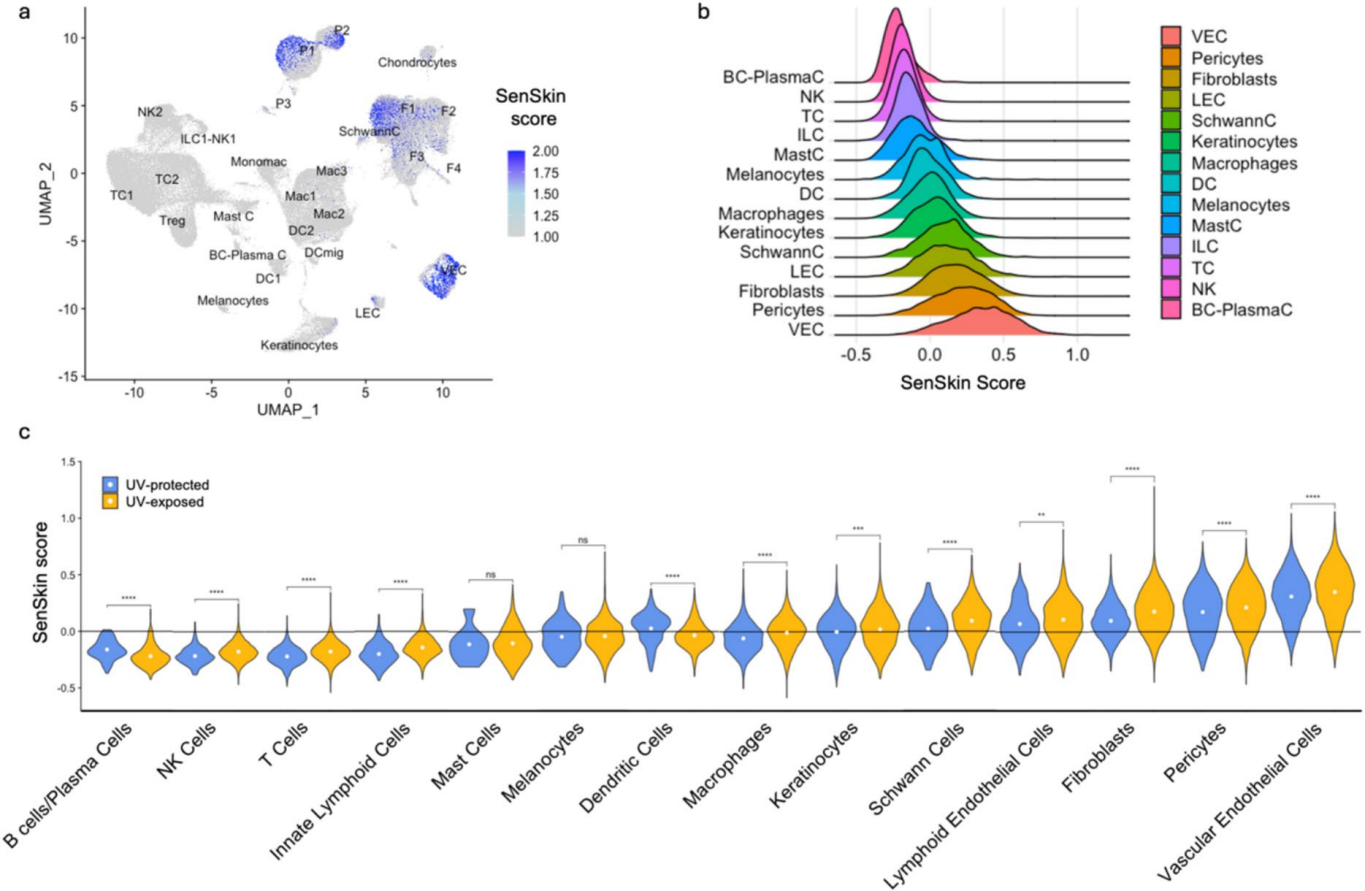
Enrichment of SenSkin™ in photoaging in scRNA-seq. **a** SenSkin™ composite score in UMAP labelled with cell types. **b** SenSkin™ composite score across skin cell types. **c**. Differences in SenSkin™ composite score in UV-exposed vs. UV-protected skin in each skin cell type

Overall, cell types with the highest SenSkin™ scores appeared to be fibroblasts, pericytes, and vascular endothelial cells (shown in Fig. 3a, b). SenSkin was significantly increased in most skin cell types in UV-exposed skin, including T cells, fibroblasts, macrophages, pericytes, NK cells, keratinocytes, vascular endothelial cells, innate lymphoid cells, Schwann cells, and lymphatic endothelial cells (shown in Fig. 3c). A few cell types—dendritic cells and B cells/plasma cells—showed a significant decrease in SenSkin™, which could be attributed to the complexity of immune aging, together with bidirectional relationships between senescent and immune cells. This is especially important given the large number of immune-related genes in SenSkin™. No significant differences were detected in mast cells or melanocytes with UV exposure, which may be attributed to the relatively small size of their populations.

## Discussion

Although multiple general, non-tissue-specific cellular senescence gene sets have been curated, senescent cell phenotypes and markers have been demonstrated to vary by tissue and organ type. Skin-specific or, generally, tissue-specific cellular senescence gene sets have not yet become widely available or applied. SenSkin™ could serve as a model for developing tissue-specific gene sets, as it specifically included genes with evidence that supported the validity of the gene in the tissue of interest. Moreover, these findings suggest that tissue-specific gene sets could be more effective than non-tissue-specific gene sets, as SenSkin™ was more enriched in chronologically aged skin than any of the other gene sets tested.

Although this study is limited by the sample size and diversity of the datasets employed, future studies could examine the role of various factors on SenSkin™, such as sex, race, and/or skin color. SenSkin™ could also be applied to examine the role of cellular senescence in a variety of skin conditions and pathologies, such as inflammatory skin diseases, skin cancers, or wounds. Genes included in SenSkin™ are limited by existing information involving senescence markers in the skin. To that end, SenSkin™ could be updated as compelling evidence for or against specific genes in senescent skin cells arises. Although SenSkin™ addresses the need for a skin-specific cellular senescence gene set, the heterogeneity of cellular senescence phenotypes in different cell types could render SenSkin™ more useful in some cell types than others. Tissue-specific or even cell type-specific cellular senescence gene sets could enhance the identification of senescent cells across the body and improve the detection and analysis of senescent cells in various conditions.

SenSkin™ could potentially be valuable as a predictive marker for senotherapeutic, or cellular senescence-targeting, agents in the future. The next steps include evaluating the sensitivity of SenSkin™ across varied diseases involving the skin, thus implicating cellular senescence, as well as its sensitivity for detecting changes following interventions. If SenSkin™ is a robust marker of cellular senescence in skin disease, aging, and interventions, it could serve as a valuable tool for the translation and regulation of senotherapeutics.

## Supplementary Information

Below is the link to the electronic supplementary material.

Supplementary file1 (DOCX 21674 KB)

## Data Availability

No new datasets were generated in this study. All datasets used are publicly available.
