# Supplementary material for: SenSkin™: a human skin-specific cellular senescence gene set
Source: GeroScience. 2025 Feb 25;47(3):2631–8. doi: 10.1007/s11357-025-01568-y (PMC12181445; doi:10.1007/s11357-025-01568-y)
Supplement: Supplementary file 1 — Supplementary file1 (DOCX 21674 KB) [file 11357_2025_1568_MOESM1_ESM.docx]

**Supplementary Information - *SenSkin^TM^: A Human Skin-Specific Cellular Senescence Gene Set***


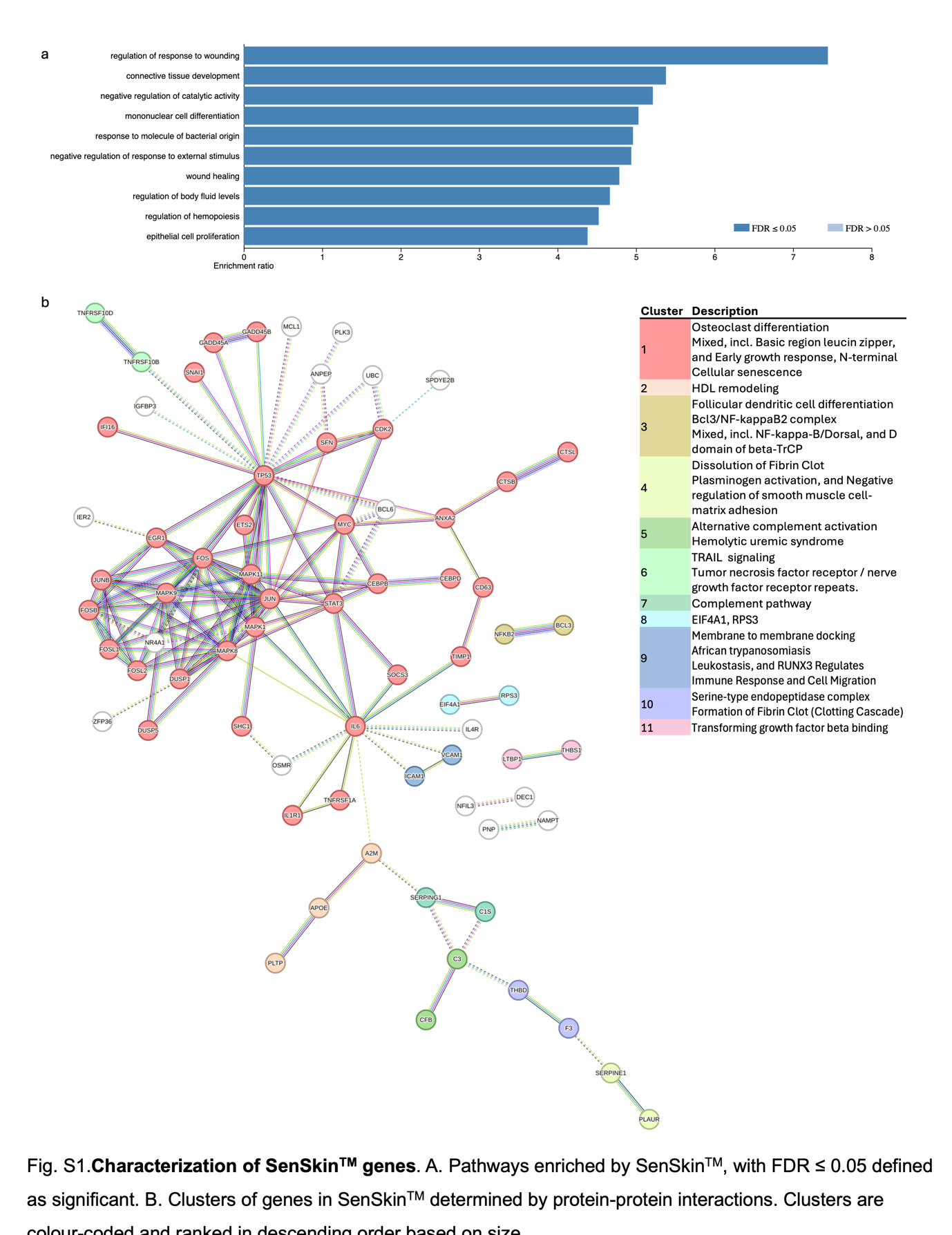


**Fig. S1 Characterization of SenSkin^TM^ genes.** a. Pathways enriched by SenSkin^TM^, with FDR ≤ 0.05 defined as significant. b. Clusters of genes in SenSkin^TM^ determined by protein-protein interactions. Clusters are colour-coded and ranked in descending order based on size


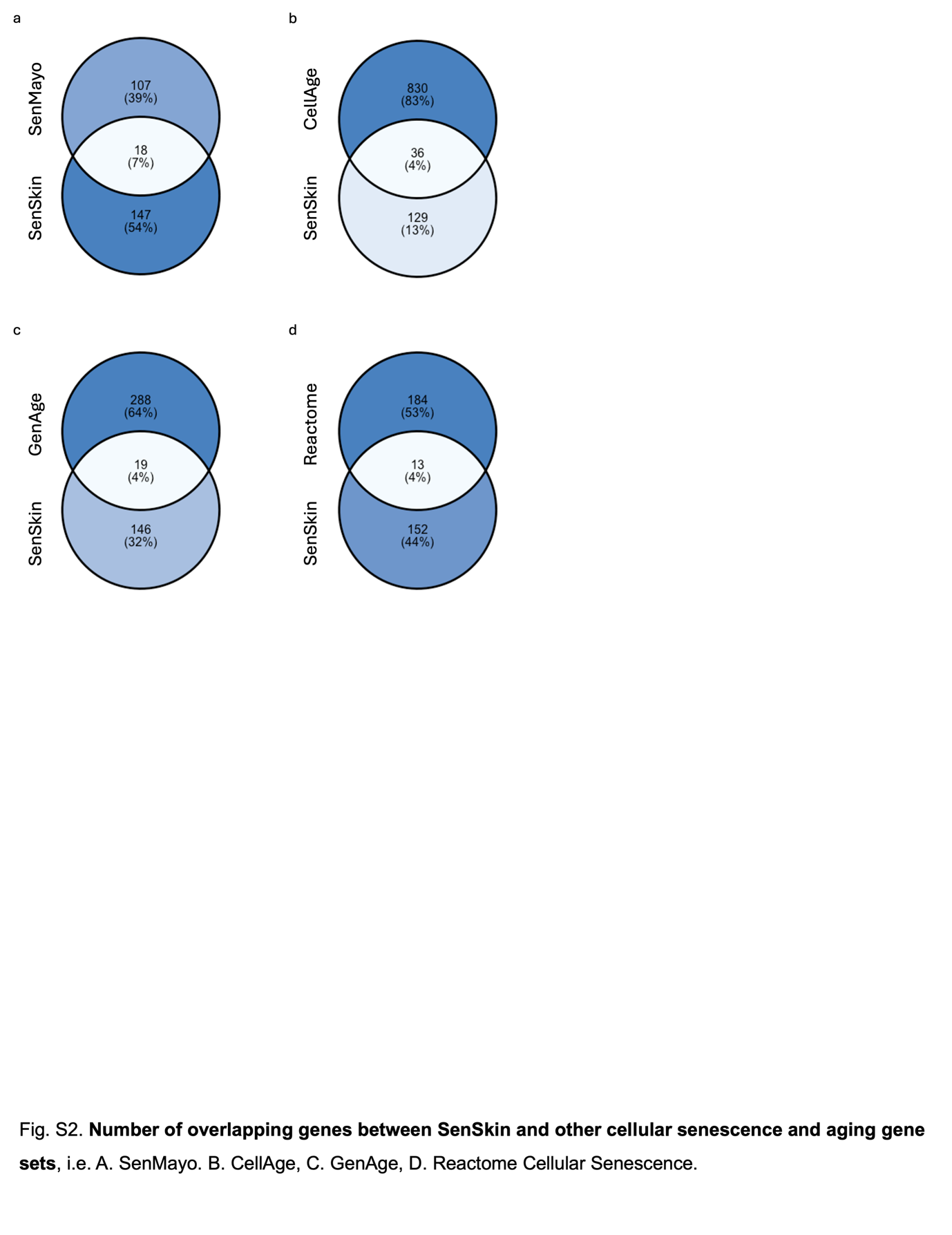


**Fig. S2** **Number of overlapping genes between SenSkin and other cellular senescence and aging gene sets**, *i.e.* a. SenMayo. b. CellAge, c. GenAge, d. Reactome Cellular Senescence


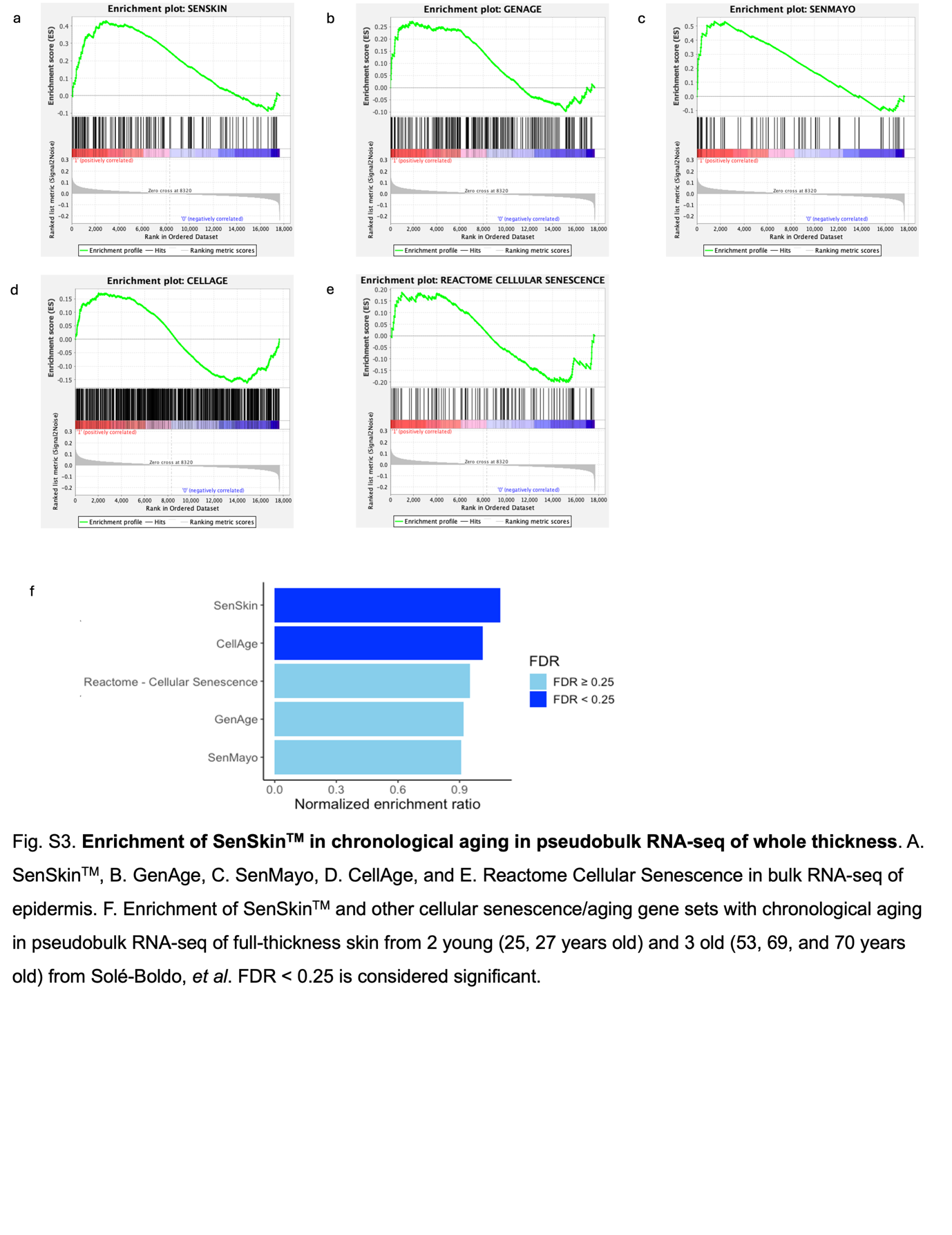


**Fig. S3**  **Enrichment of SenSkin^TM^ in chronological aging in pseudobulk RNA-seq of whole thickness.** a. SenSkin^TM^, b. GenAge, c. SenMayo, d. CellAge, and e. Reactome Cellular Senescence in bulk RNA-seq of epidermis. f. Enrichment of SenSkin^TM^ and other cellular senescence/aging gene sets with chronological aging in pseudobulk RNA-seq of full-thickness skin from 2 young (25, 27 years old) and 3 old (53, 69, and 70 years old) from Solé-Boldo, *et al*. FDR < 0.25 is considered significant
